# Supplementary material for: Low-frequency neural activity reflects rule-based chunking during speech listening
Source: eLife. 2020 Apr 20;9:e55613. doi: 10.7554/eLife.55613 (PMC7213976; doi:10.7554/eLife.55613)
Supplement: Supplementary file 1. [file elife-55613-supp1.docx]

**Supplementary File 1**

The effect size of response power at 1 Hz

| condition | *d* | power |
| --- | --- | --- |
| Alternating-order sequence (*same category*) | 2.14 | 0.99 |
| Alternating-order sequence (*different category*) | 1.90 | 0.99 |
| Random-order sequence (*same category*) | 1.78 | 0.99 |
| Random-order sequence (*different category*) | 2.15 | 0.99 |
